# Identification of SOX6 and SOX12 as Prognostic Biomarkers for Clear Cell Renal Cell Carcinoma: A Retrospective Study Based on TCGA Database

**DOI:** 10.1155/2021/7190301

**Published:** 2021-11-26

**Authors:** Xiao Lyu, Xi Zhang, Li-bin Sun, Xiao-ming Cao, Xu-hui Zhang

**Affiliations:** ^1^Department of Urology, The First Hospital of Shanxi Medical University, Taiyuan, Shanxi, China; ^2^Department of Urological Surgery, Shanxi Medical University, Taiyuan, Shanxi, China

## Abstract

**Background:**

The SOX gene family has been proven to display regulatory effects on numerous diseases, particularly in the malignant progression of neoplasms. However, the molecular functions and action mechanisms of SOX genes have not been clearly elucidated in clear cell renal cell carcinoma (ccRCC). We aimed to explore the expression status, prognostic values, clinical significances, and regulatory actions of SOX genes in ccRCC.

**Methods:**

RNA-sequence data and clinical information derived from The Cancer Genome Atlas (TCGA) database was used for this study. Dysregulated SOX genes between the normal group and ccRCC group were screened using the Wilcoxon signed-rank test. The Kaplan-Meier analysis and univariate Cox analysis methods were used to estimate the overall survival (OS) and disease-specific survival (DSS) differences between different groups. The independent prognostic factors were identified by the use of uni- and multivariate assays. Subsequently, the Wilcoxon signed-rank test or Kruskal-Wallis test and the chi-square test or Fisher exact probability methods were employed to explore the association between clinicopathological variables and SOX genes. Finally, CIBERSORT was applied to study the samples and examine the infiltration of immune cells between different groups.

**Results:**

Herein, 12 dysregulated SOX genes in ccRCC were screened. Among them, two independent prognostic SOX genes (SOX6 and SOX12) were identified. Further investigation results showed that SOX6 and SOX12 were distinctly associated with clinicopathological features. Furthermore, functional enrichment analysis revealed that SOX6 and SOX12 were enriched in essential biological processes and signaling pathways. Finally, we found that the SOX6 and SOX12 expression levels were correlated with tumor-infiltrating immune cells (TIICs).

**Conclusion:**

The pooled analyses showed that SOX6 and SOX12 could serve as promising biomarkers and therapeutic targets of patients with ccRCC.

## 1. Introduction

Clear cell renal cell carcinoma (ccRCC) is the most common aggressive kidney malignant tumor, whose incidence is increasing year by year [1]. In spite of great advances in cancer treatments, the prognosis of advanced ccRCC is still poor. Currently, the reliable prognostic biomarkers for ccRCC are still limited [2, 3]; novel robust diagnostic or prognostic biomarkers are needed to improve patient prognostication.

The SOX gene family, containing more than 20 family members, are thought to be involved in diverse biological processes; deregulation of the SOX gene can disrupt gene expression, and this has been linked to many diseases, particularly in cancer progression [4]. Emerging evidence indicated that the SOX family exhibited regulatory functions in tumor development, including tumor growth, altered consistency of cancer microenvironment, and invasion [5–7]. Several SOX genes were proven to be able to screen several tumor specimens and predict clinical outcome of tumor patients. For instance, SOX1 has been proven to display a regulatory function in the invasion and migration of gastric cancer cells [8]. Elevated SOX2 expressions were involved in enhanced stemness of various neoplasms and acquired resistance to chemotherapeutic agents [9, 10]. In breast cancer, upregulation of SOX3 suppressed proliferation and stimulate apoptosis of cancer cells [11]. The SOX4 gene was elevated in >22 types of malignant tumors, and it is widely considered as an oncogene [12]. In gastric cancer, hypoxia could lead to the activation of SOX5/Wnt pathway via inhibiting miRNA-338-3p, subsequently affecting tumor risk [13]. In general, it has been demonstrated that the SOX gene family played critical roles in the pathogenesis of various cancers. Exploring the expression patterns, prognostic values, clinical significances, and action mechanisms of the SOX gene in ccRCC offers new opportunities for targeted therapies of ccRCC. In the current study, we analyzed TCGA database to explore the role of SOX genes in ccRCC.

## 2. Materials and Methods

### 2.1. Data Collection

RNA-seq data and clinical information were obtained from TCGA (https://portal.gdc.cancer.gov/) [14], including 530 ccRCC samples and 72 noncancerous samples' transcriptome data and detailed clinical information of the corresponding patients (Table 1).

### 2.2. Identification of Dysregulated SOX Genes

The “limma” R package [15] and Wilcoxon test were applied for the preliminary identification of the dysregulated SOX genes in tumor specimens. *p* < 0.05 was considered as differentially expressed. The “pheatmap” R package was utilized to draw the heat map of the dysregulated SOX genes.

### 2.3. Survival Analysis and Uni- and Multivariate Assays

To evaluate the prognostic value of these dysregulated SOX genes, OS and DSS were estimated using the univariate Cox analysis and Kaplan-Meier (KM) analysis methods. The SOX genes significantly associated with OS and DSS were considered as prognosis-related SOX genes. Then, combined with the RNA-seq data and clinical features, the independent prognostic SOX genes and clinical variables were determined using Cox assays. These independent prognostic SOX genes were regarded as candidate SOX genes.

### 2.4. Functional Enrichment Analysis

To delve into the action mechanisms of the candidate SOX genes, we divided all samples into high- and low-expression subgroups based on the median value of the gene expression. Then, the differentially expressed genes (DEGs) between two subgroups were confirmed by the use of the “limma” R package. Genes that meet the following criteria were considered as DEGs: the absolute value log2FoldChange (FC) is greater than one, and FDR is <0.05. Metascape (http://metascape.org) was applied to study function and pathway enrichment analyses of DEGs [16].

### 2.5. Evaluation of Tumor-Infiltrating Immune Cells

TIIC proportions of ccRCC samples were evaluated using the CIBERSORT algorithm [17]. Then, the TIIC proportions of tumor samples were divided into two subgroups (high and low) and visualized by the use of violin plots.

### 2.6. Statistical Analysis

All statistical analyses were carried out using R 3.6.1 software. The OS and DSS were estimated using Cox regression assays and the Kaplan-Meier method. Univariate and multivariate assays were carried out to identify the independent prognostic SOX genes and clinical features. The chi-square test or Fisher methods were employed to explore the association between clinicopathological variables and the candidate SOX genes. *p* < 0.05 was regarded as statistically significant.

## 3. Results

### 3.1. Identification of Dysregulated SOXs

**Figure 1:**
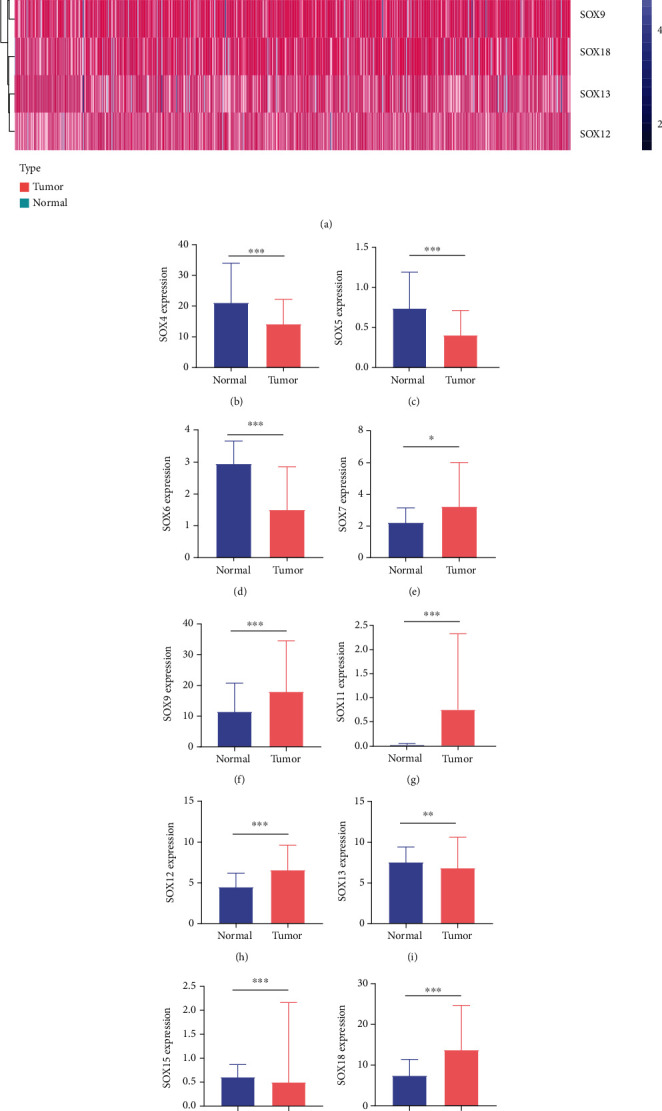
Dysregulated SOX genes in ccRCC: (a) heat map of all dysregulated SOX gene expression; (b) SOX4; (c) SOX5; (d) SOX6; (e) SOX7; (f) SOX9; (g) SOX11; (h) SOX12; (i) SOX13; (j) SOX15; (k) SOX18; (l) SOX21; (m) SOX30.

Using the “limma” R package and Wilcoxon signed-rank test, a total of 12 dysregulated SOX genes in ccRCC were identified.  Figure 1(a) revealed the pheatmap of all differentially expressed SOXs. Among them, SOX4/5/6/13/15/30 were distinctly lower in ccRCC specimens than in noncancerous specimens (Figures 1(b)–1(d), 1(i), 1(j), and 1(m)), while SOX7/9/11/12/18/21 were highly expressed in ccRCC compared with nontumor specimens (Figures 1(e)–1(h), 1(k), and 1(l)).

### 3.2. Identification of Prognosis-Related SOX Genes

**Figure 2:**
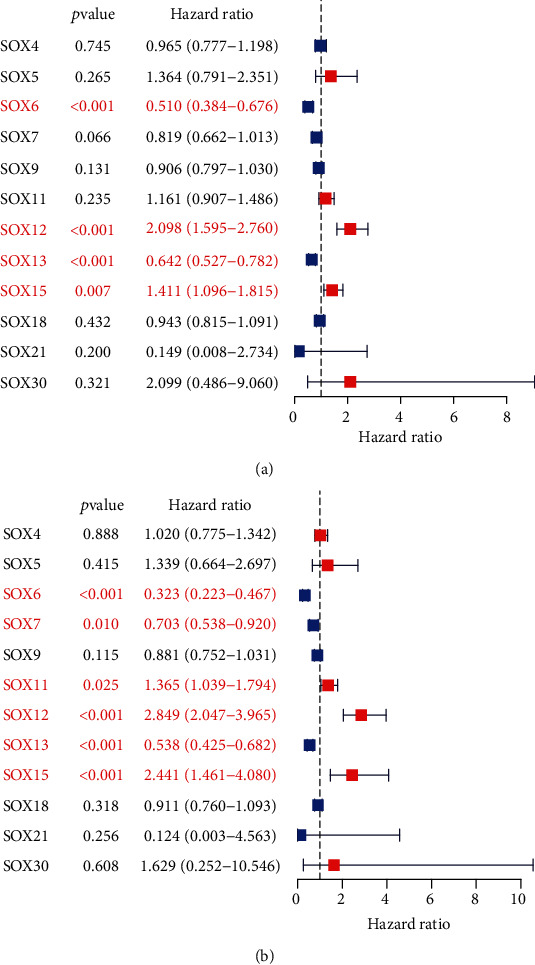
Survival analysis of SOX genes in ccRCC: (a) univariate analyses for overall survival; (b) univariate analyses for disease-specific survival.

**Figure 3:**
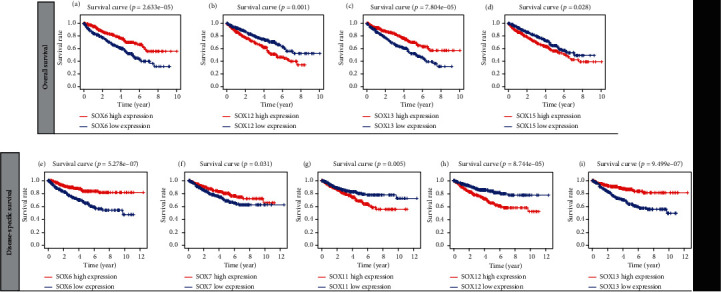
KM curves of overall survival for SOX6 (a), SOX12 (b), SOX13 (c), and SOX15 (d); KM curves of disease-specific survival for SOX6 (e), SOX7 (f), SOX11 (g), SOX12 (h), and SOX13 (i).

Using the Cox regression analysis method, we found that SOX6 and SOX13 were protective factors for patients' OS (hazard ratio (HR) < 1), while SOX12 and SOX15 were risk-associated factors (HR > 1) (Figure 2(a)). For patients' DSS, SOX6, SOX7, and SOX13 were protective factors, whereas SOX11, SOX12, and SOX15 were risk-associated factors (Figure 2(b)). Using the Kaplan-Meier method, we noticed that high SOX12 and SOX15 expression indicated worse OS, while high SOX6 and SOX13 expression suggested favorable OS (Figures 3(a)–3(d)). In terms of DSS, Kaplan-Meier survival curves showed that low SOX6, SOX7, and SOX13 expression indicated adverse DSS (Figures 3(e), 3(f), and 3(i)), while high SOX11 and SOX12 expression indicated worse DSS (Figures 3(g) and 3(h)). Collectively, the above results showed that SOX6 and SOX13 were protective factors for patients' OS and DSS, low SOX6 and SOX13 expression predicted worse prognosis of patients, while SOX12 was risk-associated factor patients' OS and DSS, and high SOX12 expressions were remarkably associated with adverse prognosis of patients.

### 3.3. Identification of Independent Prognosis Factors

**Figure 4:**
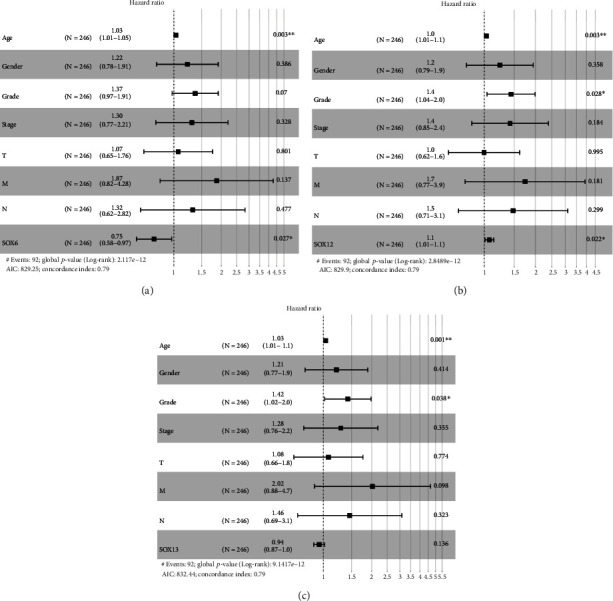
Forest plots of the results of multivariate assays of distinct prognostic factors: SOX6 (a), SOX12 (b), and SOX13 (c). Note: ^∗^*p* < 0.05; ^∗∗^*p* < 0.01.

As shown in Table 2, in univariate analysis, SOX6 expression (HR = 0.588, *p* < 0.001), SOX12 expression (*p* < 0.001), and SOX13 expression (*p* < 0.01) were associated with poorer OS of ccRCC patients. Moreover, multivariate assays suggested that SOX6 expression (HR = 0.75, *p* < 0.05) and SOX12 expression (HR = 1.10, *p* < 0.05) of patients with ccRCC were independently associated with shorter OS (Figures 4(a) and 4(b)). However, no statistical difference was found for SOX13 (Figure 4(c)).

### 3.4. Association of SOX6 and SOX12 Expression with Clinicopathological Features

**Figure 5:**
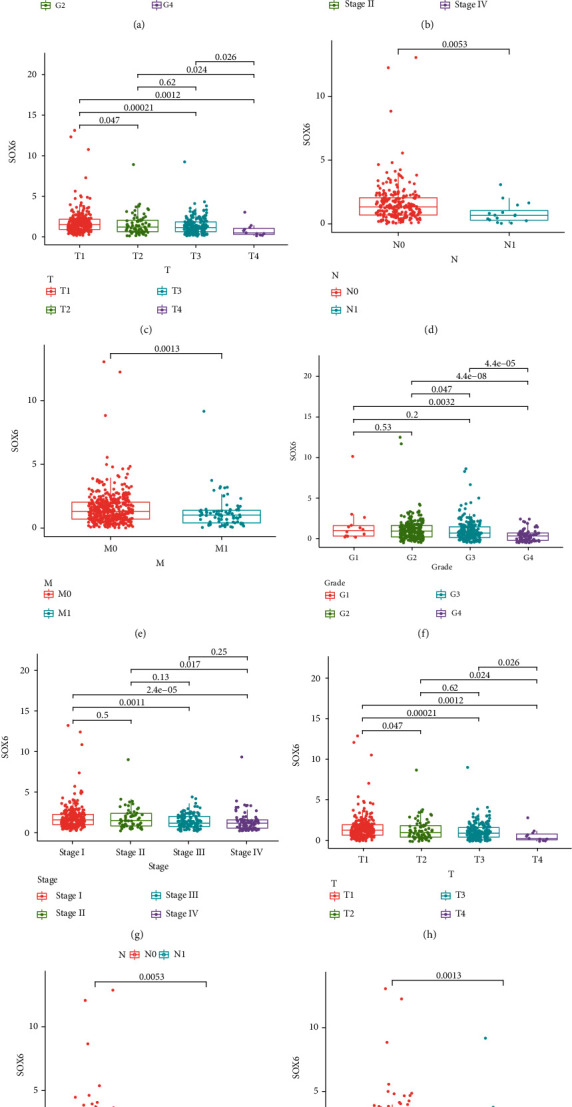
The associations between SOX6/SOX12 expressions and clinicopathological factors. (a, f) Histological grade; (b, g) clinical stage; (c, h) T status; (d, i) N status; (e, j) M status. Note: ^∗^*p* < 0.05; ^∗∗^*p* < 0.01; ^∗∗∗^*p* < 0.001.

As revealed in Figures 5(a)–5(e), low SOX6 expressions were distinctly associated with worse histological grade, advanced clinical stage, and TNM status, while upregulated expression of SOX12 predicted worse histological grade, advanced clinical stage, T status, and M status (Figures 5(f)–5(h) and 5(j)). However, for N status, the result was not statistically significant (Figure 5(i)). The chi-square test was applied to further delve into the relationships between SOX6 and SOX12 levels with clinical features. As shown in Table 3, the higher the histological grade, M status, T status, and clinical stage, the lower the SOX6 expression level, while the high expression level of SOX12 indicated advanced histological grade, M status, T status, and clinical stage (Table 4).

### 3.5. Identification of Potential Molecular Mechanisms

**Figure 6:**
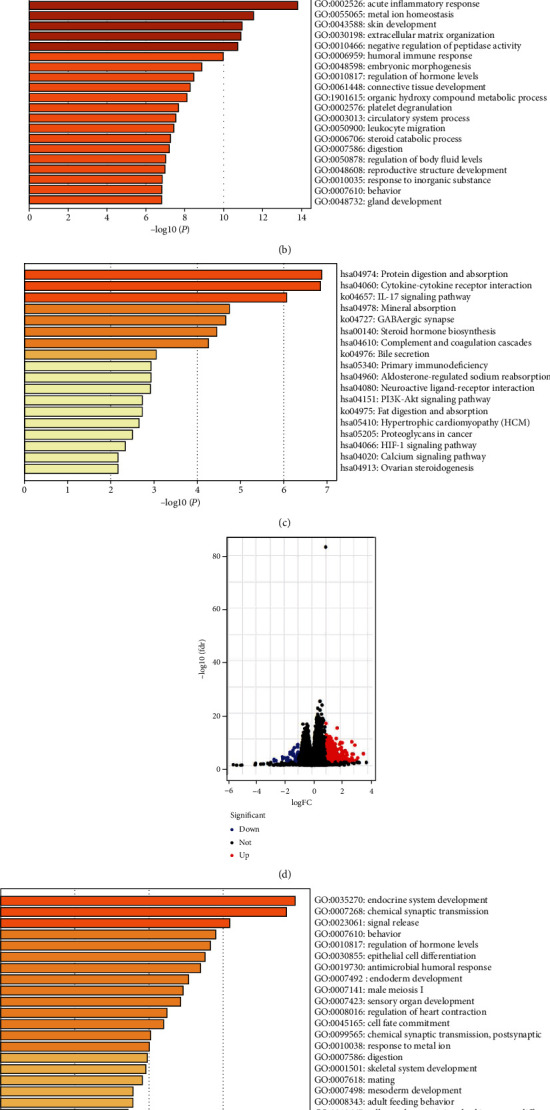
Functional enrichment analysis: (a) the DEGs between high- and low- SOX6 expression subgroups; (b) biological processes (BP) analysis of DEGs between high and low SOX6 expression groups; (c) KEGG analysis of DEGs between high and low SOX6 expression groups; (d) the DEGs between high- and low- SOX12 expression subgroups; (e) biological processes (BP) analysis of DEGs between high and low SOX12 expression groups; (f) KEGG analysis of DEGs between high and low SOX12 expression groups.

To explore the action mechanisms of SOX6/12, we identified DEGs between SOX6/12 high- and low-expression subgroups based on the mean expression of SOX6/12 in all samples.  Figure 6(a) showed the volcano plots of DEGs based on SOX6 expressions. Then, we performed functional enrichment assays of these DEGs to search out the potential biological processes (BP) and signaling pathways. In terms of BP, SOX6 was mainly involved in the acute inflammatory response, metal ion homeostasis, skin development, extracellular matrix organization, and negative regulation of peptidase activity (Figure 6(b)). KEGG pathway analysis showed that SOX6 was significantly associated with several classical cancer-related signaling pathways, such as cytokine-cytokine receptor interaction, IL-17 pathway, HIF-1 signaling pathway, proteoglycans in cancer, and PI3K-Akt signaling pathway (Figure 6(c)).  Figure 6(d) showed the volcano plots of DEGs based on SOX12 expressions. SOX12 was mainly involved in endocrine system development, chemical synaptic transmission, signal release, behavior, and regulation of hormone levels (Figure 6(e)). Moreover, SOX12 was remarkably enriched in neuroactive ligand-receptor interaction, cholinergic synapse, maturity-onset diabetes of the young, synaptic vesicle cycle, calcium signaling pathway, estrogen signaling pathway, cardiac muscle contraction, and steroid hormone biosynthesis (Figure 6(f)).

### 3.6. Association of SOX6 and SOX12 Expression with TIICs

**Figure 7:**
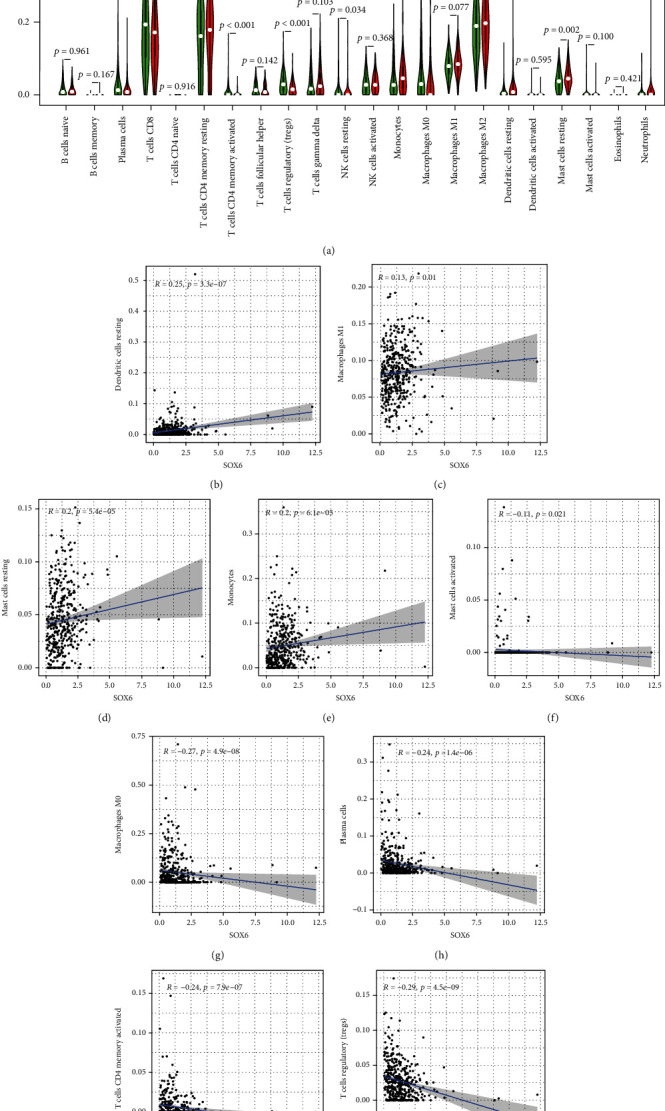
The association between SOX6 expression with tumor-infiltrating immune cells (TIICs). Difference analysis of 22 immune cell infiltration between high- and low-expression groups (a); the correlation between SOX6 expression and immune infiltration of resting dendritic cells (b), M0 macrophages (c), M1 macrophages (d), activated mast cells (e), resting mast cells (f), monocytes (g), plasma cells (h), activated memory CD4 T cells (i), and regulatory T cells (Tregs) (j).

**Figure 8:**
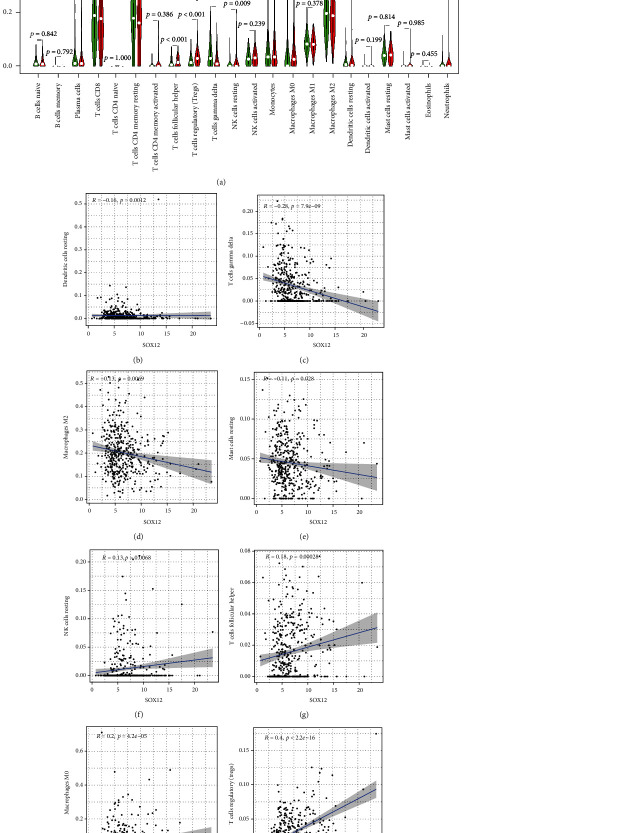
The association between SOX12 expression and TIICs. Difference analysis of 22 immune cell infiltration between high- and low-expression groups (a); the correlation between SOX12 expression and immune infiltration of resting dendritic cells (b), M0 macrophages (c), M2 macrophages (d), resting mast cells (e), resting NK cells (f), follicular helper T cells (g), gamma delta T cells (h), and regulatory T cells (Tregs) (i).

Then, our group explores the possible associations between SOX6 and SOX12 expressions with TIICs. As presented in Figure 7(a), the low SOX6 expression group has higher infiltrating levels of plasma cells (*p* < 0.001), activated memory CD4 T cells (*p* < 0.001), regulatory T cells (Tregs) (*p* < 0.001), resting NK cells (*p* < 0.05), and M0 macrophages (*p* < 0.001) than the high-expression group, while the high SOX6 expression group has higher infiltrating levels of monocytes (*p* < 0.01), resting dendritic cells (*p* < 0.001), and resting mast cells (*p* < 0.01) than the low-expression group. Correlation analysis showed that SOX6 was positively associated with resting dendritic cells (Figure 7(b)), M1 macrophages (Figure 7(c)), resting mast cells (Figure 7(d)), and monocytes (Figure 7(e)), while SOX6 expression was negatively correlated with activated mast cells (Figure 7(f)), M0 macrophages (Figure 7(g)), plasma cells (Figure 7(h)), activated memory CD4 T cells (Figure 7(i)), and regulatory T cells (Tregs) (Figure 7(j)). In terms of SOX12, as shown in Figure 8(a), the high-expression group has higher infiltrating levels of follicular helper T cells, regulatory T cells (Tregs), M0 macrophages, and resting NK cells than the low-expression group, whereas the low SOX12 expression group has higher infiltrating levels of gamma delta T cells and resting dendritic cells than the high-expression group. Correlation analysis showed that SOX12 expression was negatively correlated with resting dendritic cells (Figure 8(b)), gamma delta T cells (Figure 8(c)), M2 macrophages (Figure 8(d)), and resting mast cells (Figure 8(e)), while it was positively associated with resting NK cells (Figure 8(f)), follicular helper T cells (Figure 8(g)), M0 macrophages (*p* < 0.001, Figure 8(h)), and regulatory T cells (Tregs) (Figure 8(i)).

## 4. Discussion

Herein, we found that 12 SOX genes were dysregulated in ccRCC; among them, SOX4/5/6/13/15/30 were distinctly lowly expressed in ccRCC samples. SOX7/9/11/12/18/21 were highly expressed in ccRCC. Survival assays revealed that low expressions of SOX6/13 predicted a poor OS and DSS, while higher SOX12 expression indicated a worse prognosis. Uni- and multivariate assays suggested that low SOX6 or high SOX12 expression was an independent prognostic factor for poor overall survival of patients with ccRCC.

Growing evidence has demonstrated that SOX6 serve as a tumor suppressor in the onset and progression of human cancer. Chen et al. [18] demonstrated that SOX6 was downregulated in cervical cancer, and decreased SOX6 expression significantly stimulated the proliferation, migration, and invasion of tumor cells. Jiang et al. [19] showed that SOX6 was decreased in patients with pancreatic cancer, and decreased SOX6 expression could promote proliferation and metastasis of tumor cells. SOX6 downregulation was also found in prostate cancer, hepatocellular carcinoma, lung cancer, and breast cancer, while its downregulation was associated with the malignant phenotype of neoplasms [20–23]. SOX12 has been demonstrated to exhibit a regulatory function in carcinogenesis and cancer progression. Du et al. [24] indicated that SOX12 was highly expressed in colorectal cancer and its overexpression indicated a poorer prognosis of patients; mechanistically, SOX12 stimulated tumor cell proliferation and metastasis by modulating asparagine synthesis. Also, SOX12 was significantly increased in breast cancer, and overexpression of SOX12 predicted a poor clinical outcome of patients [25]. Zou et al. [26] found that SOX12 may serve as a cancer stem-like cell marker in hepatocellular carcinoma, which was associated with the chemoresistance of tumor cells. In lung cancer, SOX12 was found to be upregulated in cancerous samples, and loss of SOX12 in tumor cells suppressed proliferation, migration, and invasion in vitro but stimulated apoptosis of tumor cells [27]. Although SOX6/12 play a crucial role in carcinogenicity and progression, much remains unknown about their roles in ccRCC. In our study, SOX6 was found to be downregulated in ccRCC, while SOX12 was upregulated. The low SOX6 or high SOX12 expression indicates a poor clinical outcome and prognosis. The results were consistent with what was reported in the literature, indicating that SOX6 may serve as a tumor suppressor, while SOX12 may serve as an oncogene in ccRCC.

Then, to explore the molecular mechanisms involved in SOX6 and SOX12 in RCC, we performed function and pathway enrichment analyses between high- and low-expression groups; we found that SOX6 and SOX12 might regulate the ccRCC progression through several cancer-related signaling pathways, such as cytokine-cytokine receptor interaction, IL-17 signaling pathway, and PI3K-Akt signaling pathway. These signaling pathways might serve as key points to figure out the potential action mechanisms of SOX6/12 in RCC. Immune cells are an essential immune microenvironment component, which are closely associated with tumor occurrence and progression [28, 29]. Therefore, we estimated the association between SOX6/12 expression and TIICs in ccRCC applying the CIBERSORT algorithm for the first time. Interestingly, we found that low SOX6 expression or high SOX12 expression was distinctly associated with higher infiltrating levels of regulatory T cells (Tregs), the essential regulators of immune tolerance [30]. Our data revealed that SOX6 and SOX12 might play a specific role in the immunosuppressive tumor microenvironment by regulating regulatory T cells (Tregs).

## 5. Conclusions

Our findings validated that several SOX genes are abnormally expressed in ccRCC and distinctly associated with the outcome of ccRCC patients. Besides, SOX6 and SOX12 are expected to be the most promising therapeutic targets in ccRCC treatment.

## Figures and Tables

**Table 1:** Clinical information of clear cell renal cell carcinoma patients in The Cancer Genome Atlas (TCGA) database.

Clinical features	Variables	Total (*n* = 530)	Percentages (%)
Age	≤60	264	49.81
	>60	266	50.19

Gender	Female	186	35.09
	Male	344	64.91

Histological grade	G1	14	2.64
	G2	227	42.83
	G3	206	38.87
	G4	75	14.15
	GX	5	0.94
	Unknown	3	0.57

Clinical stage	Stage I	265	50
	Stage II	57	10.74
	Stage III	123	23.21
	Stage IV	82	15.48
	Unknown	3	0.57

T classification	T1	271	51.13
	T2	69	13.02
	T3	179	33.77
	T4	11	2.08

Distant metastasis	M0	420	79.25
	M1	78	14.72
	MX	30	5.66
	Unknown	2	0.37

Lymph nodes	N0	239	45.09
	N1	16	3.02
	NX	275	51.89

**Table 2:** Hazard ratio (HR) and 95% confidence interval (CI) were measured by univariate Cox regression analysis.

ID	HR	HR.95L	HR.95H	*p* value
Age	1.023	1.005	1.041	∗
Gender	1.013	0.666	1.541	0.9511
Grade	2.242	1.682	2.988	∗∗∗
Stage	1.862	1.541	2.251	∗∗∗
T	1.943	1.538	2.456	∗∗∗
M	4.073	2.634	6.300	∗∗∗
N	2.932	1.516	5.668	∗∗
SOX6	0.588	0.455	0.760	∗∗∗
SOX12	1.113	1.054	1.175	∗∗∗
SOX13	0.901	0.842	0.964	∗∗

^∗^ *p* < 0.05; ^∗∗^*p* < 0.01; ^∗∗∗^*p* < 0.001.

**Table 3:** The association between SOX6 expression and clinicopathologic factors.

Clinical features	Type	Total	High	Low	*p* value
Age	>60	266 (50.19%)	128 (48.3%)	138 (52.08%)	0.4343
	≤60	264 (49.81%)	137 (51.7%)	127 (47.92%)	

Gender	Female	186 (35.09%)	106 (40%)	80 (30.19%)	∗
	Male	344 (64.91%)	159 (60%)	185 (69.81%)	

Stage	Stage I	265 (50.28%)	151 (57.41%)	114 (43.18%)	∗∗
	Stage II	57 (10.82%)	31 (11.79%)	26 (9.85%)	
	Stage III	123 (23.34%)	52 (19.77%)	71 (26.89%)	
	Stage IV	82 (15.56%)	29 (11.03%)	53 (20.08%)	

Grade	G1	14 (2.68%)	8 (3.07%)	6 (2.3%)	∗∗∗
	G2	227 (43.49%)	132 (50.57%)	95 (36.4%)	
	G3	206 (39.46%)	102 (39.08%)	104 (39.85%)	
	G4	75 (14.37%)	19 (7.28%)	56 (21.46%)	

T	T1	271 (51.13%)	156 (58.87%)	115 (43.4%)	∗∗
	T2	69 (13.02%)	33 (12.45%)	36 (13.58%)	
	T3	179 (33.77%)	74 (27.92%)	105 (39.62%)	
	T4	11 (2.08%)	2 (0.75%)	9 (3.4%)	

N	N0	239 (93.73%)	123 (96.85%)	116 (90.62%)	0.0732
	N1	16 (6.27%)	4 (3.15%)	12 (9.38%)	

M	M0	420 (84.34%)	221 (88.76%)	199 (79.92%)	∗∗
	M1	78 (15.66%)	28 (11.24%)	50 (20.08%)	

^∗^ *p* < 0.05; ^∗∗^*p* < 0.01; ^∗∗∗^*p* < 0.001.

**Table 4:** The association between SOX12 expression and clinicopathologic factors.

Clinical features	Type	Total	High	Low	*p* value
Age	>60	266 (50.19%)	139 (52.45%)	127 (47.92%)	0.3393
	≤60	264 (49.81%)	126 (47.55%)	138 (52.08%)	

Gender	Female	186 (35.09%)	85 (32.08%)	101 (38.11%)	0.1722
	Male	344 (64.91%)	180 (67.92%)	164 (61.89%)	

Stage	Stage I	265 (50.28%)	115 (43.73%)	150 (56.82%)	∗∗
	Stage II	57 (10.82%)	25 (9.51%)	32 (12.12%)	
	Stage III	123 (23.34%)	69 (26.24%)	54 (20.45%)	
	Stage IV	82 (15.56%)	54 (20.53%)	28 (10.61%)	

Grade	G1	14 (2.68%)	8 (3.07%)	6 (2.3%)	∗
	G2	227 (43.49%)	95 (36.4%)	132 (50.57%)	
	G3	206 (39.46%)	113 (43.3%)	93 (35.63%)	
	G4	75 (14.37%)	45 (17.24%)	30 (11.49%)	

T	T1	271 (51.13%)	119 (44.91%)	152 (57.36%)	∗∗
	T2	69 (13.02%)	32 (12.08%)	37 (13.96%)	
	T3	179 (33.77%)	107 (40.38%)	72 (27.17%)	
	T4	11 (2.08%)	7 (2.64%)	4 (1.51%)	

N	N0	239 (93.73%)	115 (90.55%)	124 (96.88%)	0.0682
	N1	16 (6.27%)	12 (9.45%)	4 (3.12%)	

M	M0	420 (84.34%)	198 (79.52%)	222 (89.16%)	∗∗
	M1	78 (15.66%)	51 (20.48%)	27 (10.84%)	

^∗^ *p* < 0.05; ^∗∗^*p* < 0.01.

## Data Availability

The authors certify that all the original data in this research could be obtained from public database. All data generated or analyzed during this study are included in this article.
